# Creating and evaluating the score to assess overdose: the OD score

**DOI:** 10.1186/s12873-023-00923-6

**Published:** 2024-01-07

**Authors:** Kazuki Nagashima, Nobuhiro Yasuno, Machiko Watanabe

**Affiliations:** 1 Laboratory of Practical Pharmacy, Graduate School and Faculty of Pharmaceutical Sciences, Chiba University, 1-8-1 Inohana, Chuo-ku, Chiba, 260-8675 Japan; 2 Laboratory of Clinical Pharmaceutics, Faculty of Pharma-Science, Teikyo University, 2-11-1 Kaga, Itabashi-ku, Tokyo, 173-8605 Japan; 3 Laboratory of Hospital Pharmacy, Faculty of Pharma-Science, Teikyo University, 2-11-1 Kaga, Itabashi-ku, Tokyo, 173-8605 Japan

**Keywords:** Emergency department, Overdose, Psychiatry, Scoring, Suicide, The OD score

## Abstract

**Background:**

During disasters (including epidemics such as coronavirus disease 2019), the capacity of emergency departments is exceeded, thereby hindering the administration of appropriate lifesaving measures. Furthermore, the number of overdose patients increases because of the stress overload during emergency situation. The fact that overdose patients are forced to be transported to medical facilities that do not typically treat them is becoming worrisome. Moreover, there is no definitive score for overdose. This study aimed to create a patient-specific scoring system to assess overdose.

**Methods:**

This was a retrospective single-center study. The evidence-based OD score was evaluated on a scale of 0–15. Further, logistic analysis and receiver operating characteristic (ROC) curve analysis were performed to evaluate the score.

**Results:**

Overall, 262 patients (including 118 overdose patients) receiving care at the intensive care unit of Japan’s Teikyo University Hospital in 2021 were targeted. Regarding the total OD score, ROC analysis revealed a cutoff of 8 (area under the curve [AUC]: 0.99, 95% confidence interval [CI]: 0.980–0.997, sensitivity: 0.95, specificity: 0.95, p < 0.05), which was considered to indicate an overdose. Of the items evaluated in the OD score, the scenario at the location of the patient’s discovery (adjusted odds ratio [AOR]: 16.8, 95% CI: 5.0–255.9, p = 0.002) and recent experience of mental anxiety (AOR: 55.7, 95% CI: 2.8–5399.5, p = 0.03) significantly predicted an overdose in multivariable logistic regression analysis. External validation revealed that the OD score could also identify overdose in patients treated in a cohort from 2022 (average cutoff: 8.6, average AUC: 1.0, p < 0.0001).

**Conclusions:**

The OD score could accurately assess overdose patients. Medical facilities that do not frequently address overdose patients will benefit from the use of this score.

## Introduction

According to the World Health Organization, suicide accounts for 700,000 deaths annually, and approximately 20% of suicides worldwide are due to self-poisoning with pesticides in low-income countries [1]. Drug overdose is also a method of committing suicide [2]. According to a previous report, the rate of hospitalization for drug overdose in Japan is 17 per 100,000, and for women aged 19–34 years, it is 40.9 per 100,000 [3].

Notably, most suicidal patients have psychiatric disorders [4]. In addition, there are numerous reports on the regular use of psychotropic medications (e.g., antipsychotics, antidepressants, benzodiazepines, mood stabilizers, etc.) [5–7], and overdose victims are typically young [3, 8]. Moreover, there have been reports of a link between alcohol consumption and suicide [9, 10]. Furthermore, because self-harm [11] or overdose is likely to recur [2, 12], it is critical to break destructive cycles. Several psychiatric interventions have been reported to help break this cycle [13]. These evidences can be assessed objectively and succinctly. However, no score could be used to accurately assess overdose with comprehensive use of these evidences.

A poisoning severity score (PSS) was proposed in 1998 for defining the severity of overdose [14], and it demonstrated some benefits in the determination of the severity [14, 15]. However, PSS involves various data points from 12 different organ systems and multiple subjective variables, such as mild hemolysis, mild hypotension, and persistent cough, reducing its inter-rater reliability [16]. Thus, its clinical use is skeptical [16]. In addition, PSS is not used to determine an overdose, but rather to determine the severity of an overdosed patient.

The coronavirus disease 2019 (COVID-19) outbreak has led to an increase in the number of overdose cases [17–19]. An overdose usually causes mild symptoms, but its management tends to consume many medical resources [20]. Moreover, overdose patients are typically transported to critical care centers. Disasters (including epidemics such as coronavirus disease 2019) may impact the lifesaving activities for other patients, including overdose patients. Moreover, if medical personnel are unfamiliar with overdose therapies or if patients are presented to a facility that is under-resourced and does not properly treat overdose patients, the patient is at a disadvantage. One of the problems associated with this is that there is no scoring system for assessing an overdose, posing difficulty for inexperienced medical professionals in judging an overdose. Diagnosis of drug overdose is almost the last after a diagnosis of exclusion.　Although the evidence of drug overdose is increasing, there is no score that completely evaluates the data and determines whether a drug overdose has occurred. Therefore, in the present study, we aimed to objectively evaluate the accumulated evidence on overdose and assess the effectiveness of the score by applying it to overdose patients. The proposed OD score includes components that can be evaluated even at hospitals that do not typically treat overdose patients as well as in COVID-19 or disaster situations. This OD score will be beneficial for appropriate overdose assessment.

## Materials and methods

### Target patients and study design

A total of 262 patients (including 118 overdose patients) presented and receiving care at the intensive care unit of Teikyo University Hospital, Japan, were targeted from January to December 2021. The 144 nonoverdose patients were those hospitalized until February 2021 to collect a similar number of overdose cases due to technical limitations of data extraction from the electronic medical records. As an exclusion criteria, those who died were excluded. The endpoints of this retrospective study were age, regular medications, history of self-harm (including overdose, wrist cuts, etc.), sex, the scenario at the location of the patient’s discovery, alcohol consumption, recent experience of mental anxiety, and history of psychiatric consultation (Table 1). Overdose patients were defined as those with a confirmed diagnosis of overdose, with other diagnoses being ruled out by the physician.

### Creating a score to assess overdose (the OD score)

The OD score and each of its items are presented in Table 1. It shows age, medications regularly taken by the patient (psychiatric drugs, such as antipsychotics, antidepressants, anticonvulsants, and benzodiazepines), history of self-harm (including overdose, wrist cuts, etc.), sex, and the scenario at the location of the patient’s discovery. In addition, the table shows whether empty drug packets and bottles were found and whether the patient collapsed in the room in addition to alcohol consumption, recent mental anxiety, and a history of psychiatric consultation. A recent mental anxiety was defined as one that occurred within 1 week before hospital admission. The total OD score was determined by adding points if the target patient agreed with the content of the items. The maximum-allowed score was 15, and the minimum score was 0.

**Table 1:** OD Score and items

OD Score items		Score
Age	≦39	2
	40-59	1
	≧60	0
Regular medication: Psychotropic drugs (antipsychotics, antidepressants, mood stabilizers, benzodiazepines)	with	2
	unclear	1
	without	0
History of self-harm (overdose, wrist cuts, etc.)	with	2
	unclear	1
	without	0
Sex	Female	1
	Male	0
The scenario at the location of the patient’s discovery (maximum 4)	Empty drug packets and bottles were recovered	3
	collapsed in the room	1
	not applicable	0
Alcohol consumption	with	1
	without	0
Recent experience of mental anxiety	with	1
	without	0
History of psychiatric consultation	with	2
	unclear	1
	without	0

The assessment items were based on the reported evidence related to suicide and overdose. The OD score was calculated based on age [3, 8], regular use of psychotropic drugs (antipsychotics, antidepressants, mood stabilizers, and benzodiazepines) [5–7], history of self-harm [2, 11, 12] (overdose, wrist cuts, etc.), sex [3, 8], alcohol consumption [9, 10], recent experience of mental anxiety [13], and a history of psychiatric consultations [21]. Age was classified based on previous reports [3, 8] and assessed considering the recent increase in overdose episodes among young people. Notably, the cases of overdose are more common in women [3, 8], self-harm has been reported tend to repeat [2, 11, 12], and excessive alcohol consumption has been reported to be associated with suicide [9, 10]. Based on these reports, we scored the overdose. In addition, as a special item in this study, we considered scenario at the location of the patient’s discovery.

### Statistical analysis

For logistic analysis and ROC curve analysis, we used JMP Pro 15 (SAS Institute Inc., NC, U.S.A.). The Youden Index method was used for calculating JMP Pro’s cutoff. p-values of < 0.05 were considered statistically significant. External validation of the OD score was conductedusing a patient population hospitalized between January and December in 2022 in the same institution. The statistical software R (The R Foundation, Vienna University of Economics and Business, Austria) was used to calculate the number required to perform the receiver operating characteristic (ROC) analysis. The required number of samples was determined based on a detection power of 0.8, a kappa of 15:1 (other patients:overdose), an area under the curve (AUC) value of 0.9 (from the obtained data), and a significance level of 0.05. The computed numbers of overdose and other samples were 7 (10 is recommended in this case) and 42, respectively. In the 2022 patient cohort, 10 overdose cases and 42 other cases were randomly selected for ROC analysis, and external validation of the OD score was performed five times.

## Results

### Patient characteristics and their regular medications

Table 2 shows the characteristics of the patients included in this study. The characteristics of the overdose patients were as follows: low average age (39.5 ± 18.1 years old), high proportion of women (81.4%), high rate of psychiatric consultation (85.6%), high rate of recent experience of mental anxiety (97.5%), and short hospital stay (5 ± 10.9 days). Other patients in the ICU, who were considered as controls, were admitted for reasons other than overdoses. The primary diagnoses included traumatic injury (22.9%), respiratory disease (14.6%), cardiovascular disease (9.7%), impaired consciousness (8.3%), infectious disease (8.3%), gastrointestinal disease (5.6%), shock (4.2%), COVID-19 (3.5%) convulsions (2.8%), and other (20.1%). Notably, compared with other patients, overdose patients tended to use benzodiazepines, antipsychotics, antidepressants, and mood stabilizers more frequently.

**Table 2:** Patient characteristics and regular medications

	Overall n=262	Overdose n=118	Other patients n=144
A. Patient characteristics			
Age (mean±SD)	52.9±22.9	39.5±18.1	63.9±20.5
Sex (n: Male/Female)	113/149	22/96	91/53
History of psychiatric consultation (n;%: with)	120; 45.8	101; 85.6	19; 13.2
Recent experience of mental anxiety (n;%: with)	124; 47.3	115; 97.5	9; 6.3
The scenario at the location of the patient’s discovery (n;%: collapsed in the room)	254; 96.9	118; 100	136; 94.4
Alcohol consumption (n;%: with)	49; 18.7	33; 28.0	16; 11.1
Hospitalization days (mean±SD)	13±22.7	5±10.9	19±27.6
COVID-19 (n;%: positive)	9; 3.4	2; 1.7	7; 4.9
B. Regular medications			
Benzodiazepines (n; %)		70; 59.3	23; 16.0
Antipsychotics (n; %)		44; 37.3	12; 8.3
Antidepressants (n; %)		41; 34.7	9; 6.3
Mood stabilizers (n; %)		17; 14.4	6; 4.2
Cardiovascular drugs (n; %)		15; 12.7	56; 38.9
Antidiabetic drugs (n; %)		8; 6.8	26; 18.1
Respiratory drugs (n; %)		6; 5.1	12; 8.3
OTC drugs (n; %)		0; 0	2; 1.4

### Logistic analysis of the OD score items for assessing overdose

In order to examine the odds ratio (OR) of the OD score items for assessing overdose, we performed logistic analysis (Table 3). Age (OR 4.5, 95% CI 3.1–6.6), regular medication (OR: 3.3, 95% CI: 2.5–4.6), history of self-harm (OR 15.6, 95% CI 8.6–31.1), and sex (OR: 7.5, 95% CI: 4.3–13.5), the scenario at the location of the patient’s discovery (OR: 11.6, 95% CI: 7.1–22.4) and alcohol consumption (OR 3.1, 95% CI 1.6–6.1) were found to be significant factors. Moreover, significant correlations were found between overdose patients and recent experience of mental anxiety (OR: 575.0, 95% CI: 175.6–2687.9) and previous psychiatric consultation (OR: 6.8, 95% CI: 4.8–10.0).

**Table 3:** Logistic analysis of the OD score items for assess overdose

	Univariate analysis		Multivariable analysis	
OD Score items	Crude OR (95%CI)	*p* value	Adjusted OR (95%CI)	*p* value
Age	4.5 (3.1-6.6)	<0.0001*	0.5 (0.03-2.67)	0.46
Regular medication: Psychotropic drugs (antipsychotics, antidepressants, mood stabilizers, benzodiazepines)	3.3 (2.5-4.6)	<0.0001*	11.1 (1.4-769.7)	0.11
History of self-harm (overdose, wrist cuts, etc.)	15.6 (8.6-31.1)	<0.0001*	3.3 (0.54-34.8)	0.24
Sex	7.5 (4.3-13.5)	<0.0001*	2.8 (0.22-47.8)	0.42
The scenario at the location of the patient’s discovery	11.6 (7.1-22.4)	<0.0001*	16.8 (5.0-255.9)	0.002*
Alcohol consumption	3.1 (1.6-6.1)	0.0007*	0.2 (0.01-2.1)	0.19
Recent experience of mental anxiety	575.0 (175.6-2687.9)	<0.0001*	55.7 (2.8-5399.5)	0.03*
History of psychiatric consultation	6.8 (4.8-10.0)	<0.0001*	2.5 (0.60-15.7)	0.24

Because the number of samples was sufficient, we performed multivariable logistic using all items, which indicated a significant correlation of overdose with the scenario at the location of the patient’s discovery (adjusted odds ratio [AOR]: 16.8, 95% CI: 5.0–255.9) and recent experience of mental anxiety (AOR: 55.7, 95% CI: 2.8–5399.5), but not with other factors.

### Prediction of overdose by the OD score

#### Degree of prediction of overdose by each item of the OD score

Table 4 shows the prediction of overdose patient based on each item. We used the Youden Index method for calculating JMP Pro’s cutoff. Each item was rated as follows: age (cutoff value: 1, AUC: 0.79, 95% CI: 0.74–0.84, sensitivity: 0.87, specificity: 0.60); regular medication (cutoff value: 2, AUC: 0.77, 95% CI: 0.71–0.81, sensitivity: 0.69, specificity: 0.78); history of self-harm (cutoff value: 1, AUC: 0.86, 95% CI: 0.81–0.9, sensitivity: 0.69, specificity: 0.78); sex (cutoff value: 1, AUC: 0.72, 95% CI: 0.67–0.77, sensitivity: 0.81, specificity: 0.63); the scenario at the location of the patient’s discovery (cutoff value: 4, AUC: 0.97, 95%CI: 0.95–0.99 sensitivity: 0.98, specificity: 0.97); and alcohol consumption (cutoff value: 1, AUC: 0.58, 95% CI: 0.54–0.63, sensitivity: 0.23, specificity: 0.89). Furthermore, recent experience of mental anxiety (cutoff value: 1, AUC: 0.96, 95% CI: 0.92–0.97, sensitivity: 0.97, specificity: 0.94) and history of psychiatric consultations (cutoff value: 1, AUC: 0.87, 95% CI: 0.82–0.91, sensitivity: 0.88, specificity: 0.85) were found to be the indicators of overdose.

**Table 4:** Degree of prediction of overdose by each items of the OD score

OD Score items	cut-off value	AUC	95%CI	sensitivity	specificity	*p* value
Age	1	0.79	0.74-0.84	0.87	0.6	<0.0001
Regular medication: Psychotropic drugs (antipsychotics, antidepressants, mood stabilizers, benzodiazepines)	2	0.77	0.71-0.81	0.69	0.78	<0.0001
History of self-harm (overdose, wrist cuts, etc.)	1	0.86	0.81-0.9	0.78	0.92	<0.0001
Sex	1	0.72	0.67-0.77	0.81	0.63	<0.0001
The scenario at the location of the patient’s discovery	4	0.97	0.95-0.99	0.98	0.97	<0.0001
Alcohol consumption	1	0.58	0.54-0.63	0.23	0.89	<0.0001
Recent experience of mental anxiety	1	0.96	0.92-0.97	0.97	0.94	<0.0001
History of psychiatric consultation	1	0.87	0.82-0.91	0.88	0.85	<0.0001

Notably, regarding the item scenario at the location of the patient’s discovery and recent experience of mental anxiety, overdose was strongly determined by its item, indicating that these items could be strongly associated with overdose.

#### Degree of prediction of overdose by total OD score

**Fig. 1:**
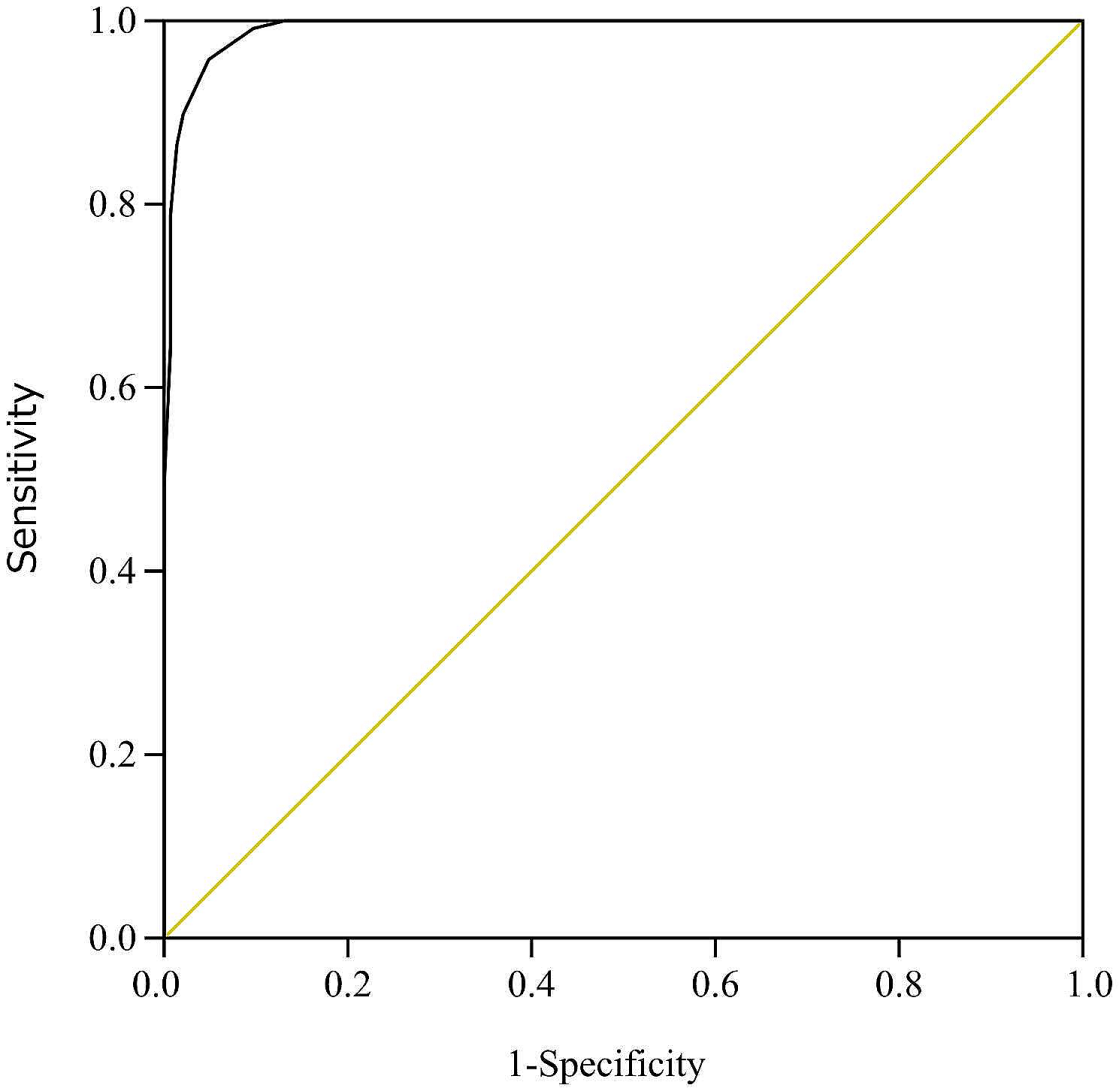
ROC curves of total OD score for the prediction of overdose. Receiver operating characteristic (ROC) analysis revealed a cutoff of 8 (area under the curve (AUC): 0.99, 95% confidence interval (CI): 0.980–0.997, sensitivity: 0.95, specificity: 0.95, p < 0.05), and was judged to be an overdose

Table 5; Fig. 1 show the ROC table and curve, respectively. Based on ROC analysis, a cutoff of 8 was considered to indicate overdose (AUC: 0.99, 95% CI: 0.980–0.997, sensitivity: 0.95, specificity: 0.95).

**Table 5:** ROC table (Total OD score)

	Total OD score	Specificity	Sensitivity	True positive	True negative	False positive	False negative
	15	1	0.0424	5	144	0	113
	14	1	0.2627	31	144	0	87
	13	1	0.4915	58	144	0	60
	12	0.9931	0.6441	76	143	1	42
	11	0.9931	0.7881	93	143	1	25
	10	0.9861	0.8644	102	142	2	16
	9	0.9792	0.8983	106	141	3	12
Cut-off value	8	0.9514	0.9576	113	137	7	5
	7	0.9028	0.9915	117	130	14	1
	6	0.8681	1	118	125	19	0
	5	0.8125	1	118	117	27	0
	4	0.6875	1	118	99	45	0
	3	0.5208	1	118	75	69	0
	2	0.2361	1	118	34	110	0
	1	0.0069	1	118	1	143	0
	0	0	1	118	0	144	0

### Assessment of overdose by total OD score

Based on the data obtained from the ROC analysis, the total OD score was determined (Table 6). This score ranged from 0 to 15, with scores of ≥ 8, 3–7, and 0–2 indicating a high, medium, and low probability of overdose, respectively.

**Table 6:** Assessment of overdose by total OD Score

Total OD score	Likelihood of drug overdose (OD)
≧8	High
3 to 7	Medium
0 to 2	Low

### External validation of the OD score

For the external validation of the OD score in a paitent population hospitalized from January to December in 2022. We randomly extracted the data from 10 overdose patients as well as 42 other patients, based on the required number of patients calculated using R, and performed ROC analysis. The external validation performed in five sets of patients revealed that the average cutoff value for the OD score to detect overdosing patients was 8.6 with an average AUC of 1.0 (p < 0.0001). In the external validation analysis, the AUC and cutoff values of the OD score were nearly identical.

## Discussion

To the best of our knowledge, this is the first study to attempt scoring patient data using the OD score to assess overdose. We found that the newly generated score could adequately assess overdose. Considering the scenario at the location of the patient’s discovery and recent experience of mental anxiety individually, there is a possibility that an overdose can be inferred from these results alone. But actually, the facts related to the scenario at the location of the patient’s discovery and elements determining whether empty drug packs and bottles were found and whether the patient collapsed in the room are probably insufficient to determine an overdose. We found that assessing each element in combination allows overdose to be reliably assessed.

According to the standards for assessing the severity and urgency of transporting overdose patients, many patients in Japan are transported to advanced medical facilities (tertiary care facilities). For example, among the top 100 causes, drug poisoning was the commonest cause in terms of the proportion of patients requiring emergency tertiary care (37.8%) [22]. The ideal way to reduce medical costs is to determine the OD score during transport; however, patient information is limited during transport. Although overdose patients generally have minor injuries, treating them consumes many medical resources [20]. In addition to caffeine as well as anticonvulsant and antipsychotic overdoses, there are severe cases in which intratracheal intubation and dialysis are critical for therapy [23–25]. However, the average length of hospital stay for overdose patients is less than 3 days [20]. Although it is widely assumed that such patients are discharged quickly because modern emergency medical facilities provide prompt care, careful consideration must be given when selecting medical facilities to ensure the effective use of medical resources. To overcome this problem, a score that can determine overdose before hospital, such as during the ambulance ride to the hospital, is required. Currently, whether the OD score can determine overdose before the hospital is a future issue. The OD score is also useful for medical professionals unfamiliar with overdose patients, even during the spread of COVID-19, normal times, and when emergency facilities are unavailable during disasters, resulting in adequate saving of patient’s lives.

Psychiatric evaluation is advised for the treatment of self-harm, including overdose, according to the guidelines in UK [26]. Notably, self-harm is associated with psychiatric problems according to a previous study [27]. However, overdose patients may present with mild symptoms, and they frequently want to be discharged against medical advice. Most overdose patients have psychiatric problems [21], and a psychiatrist’s diagnosis is important for several reasons, including the possibility of discharge. Moreover, psychiatric follow-up has been shown to be beneficial in preventing overdose recurrence [13]. However, overdose patients who are transported to hospitals without psychiatric units refrain from speaking to a psychiatrist after being discharged [20]. Furthermore, overdose tends to recur [12]. Therefore, there is a risk that overdosed patients who require hospitalization in a psychiatric unit will be missed and overdosed again. As a help, the scores in this OD score for judging overdose, “recent experience of anxiety”, and “past psychiatric consultations” can assess by medical personnel. Recent overdoses were defined as those that occurred within 1 week before hospital admission. After admission, the patient in the present study was interviewed by medical personnel; according to the electronic medical records of this study, a psychiatrist was often in charge of the interview, but in some cases, other medical personnel interviewed the patient. In particular, when suicidal ideation is imminent, psychiatric admission is generally required. Notably, Suicidal Thoughts Questionnaire–JR for pediatrics [28], the suicide intent scale [29], and Columbia-Suicide Severity Rating Scale [30] are the scores used to evaluate suicidal ideation. These scores should be evaluated calmly after recovery from the impaired consciousness due to the overdose. The patient should not be discharged without prior transfer to a psychiatric hospital if the OD score indicates the possibility of drug overdose and the facility does not have a psychiatric unit.

The OD score can be used to determine overdose; however, interventions aimed at preventing overdose are also important [31]. Because overdose is likely to recur [2, 12], it is also important to implement measures to prevent future overdose.

## Limitation

A limitation of this research is that it was single-center research. However, patient’s resemblances across all countries is the administration of medications for mental health before overdose [32–34]. Conveniently, the components of this score can be evaluated worldwide, in both developed and developing countries, and further studies in other countries can be warranted. However, the OD score does not assess severity, it only assesses overdose. As the severity of an overdose is likely to depend on the individual drug overdose rather than on the regular use of drugs, identifying the drug received by the patient is crucial [25]. The OD score of 8 indicates a high probability of overdose but does not always predict severity. The use of PSS is being considered, but it is necessary to understand the associated limitations and use the score correctly [16]. Verification of whether the OD score can select the candidate of medical institution is a future issue, the OD score only may not be suitable for selecting the facility. On the other hand, the OD score is critical to avoid overlooking overdose patients transported to hospitals that do not routinely treat OD, during epidemics such as COVID-19 and disasters. Physicians who diagnosed overdose in this study were tertiary emergency physicians familiar with overdose diagnosis and treatment. More research is needed to determine if other medical professionals, including physicians in other specialties, can identify similar findings. The OD score can be utilized for training resident doctors in addition to its usage during disasters.

## Conclusion

To the best of our knowledge, the OD score developed in this study is the first to assess overdose, and a score of ≥ 8 accurately evaluated overdose patients.

## Data Availability

The datasets that are either or both used and analyzed during the current study are available on reasonable request to corresponding author.
